# Supplementary figures and images for: Protective effect conferred by prior infection and vaccination on COVID-19 in a healthcare worker cohort in South India
Source: PLoS One. 2022 May 20;17(5):e0268797. doi: 10.1371/journal.pone.0268797 (PMC9122209; doi:10.1371/journal.pone.0268797)

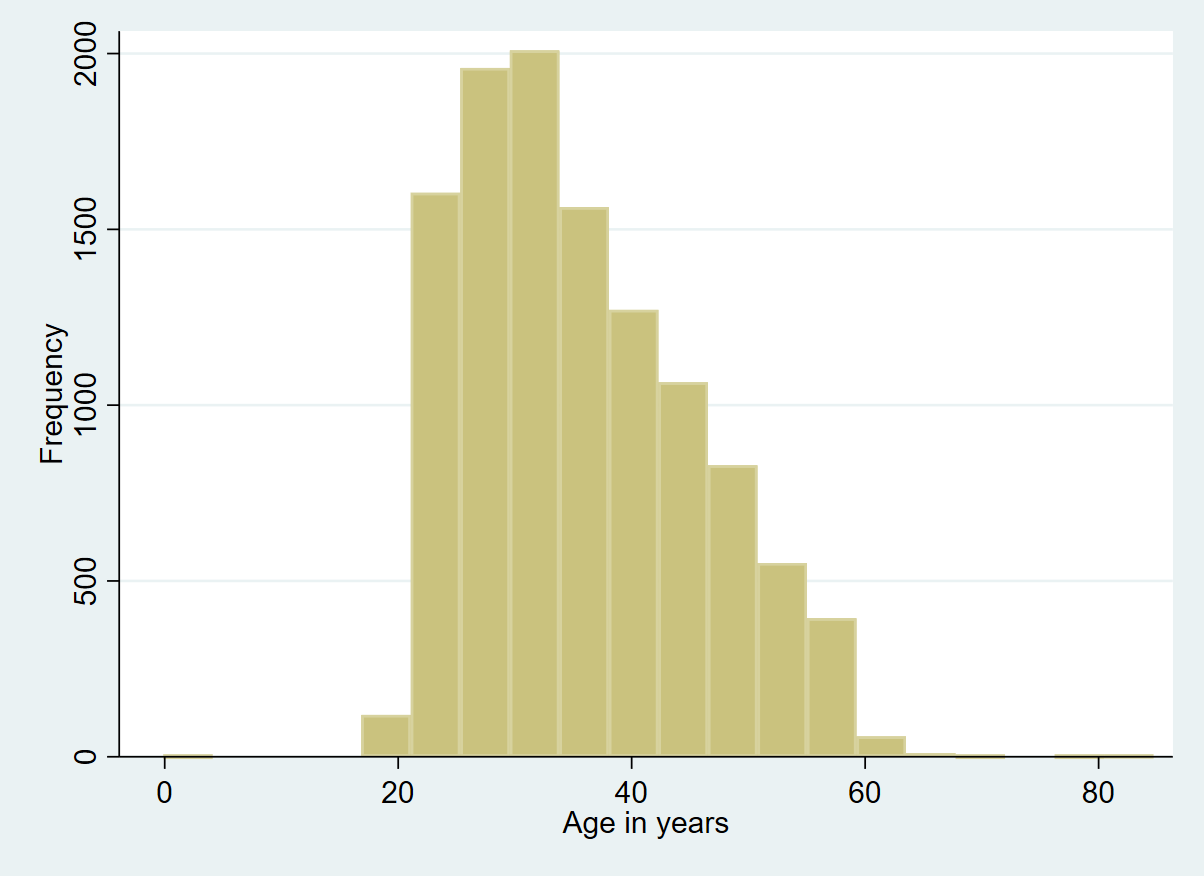

Supplement: S1 Fig — (TIF) [file pone.0268797.s001.tif]

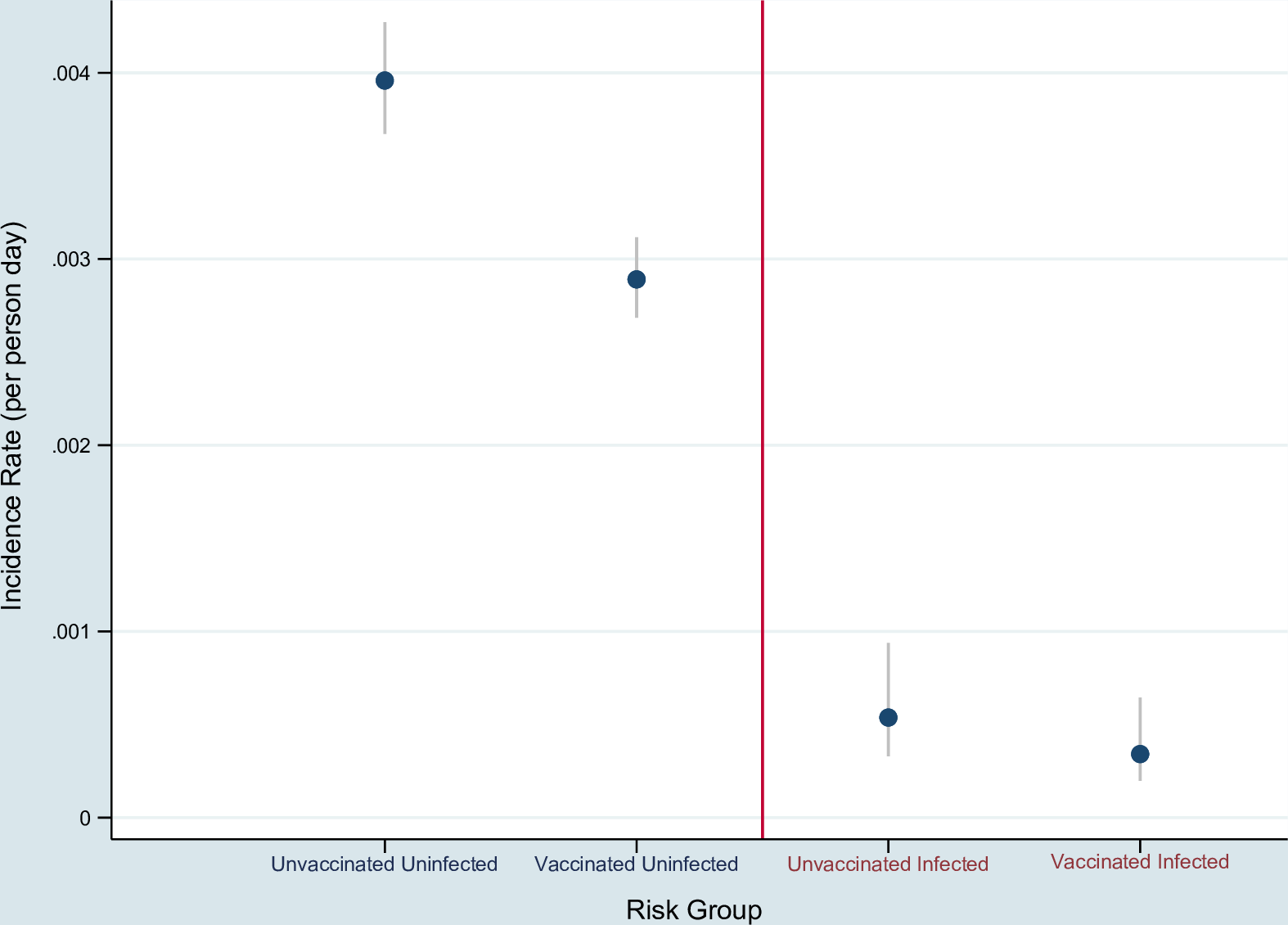

Supplement: S2 Fig — (TIF) [file pone.0268797.s002.tif]
