# Supplementary material for: Protective effect conferred by prior infection and vaccination on COVID-19 in a healthcare worker cohort in South India
Source: PLoS One. 2022 May 20;17(5):e0268797. doi: 10.1371/journal.pone.0268797 (PMC9122209; doi:10.1371/journal.pone.0268797)
Supplement: S1 Table — (DOCX) [file pone.0268797.s003.docx]

**S1 Table: Cox-proportional hazards (PH) model**

| **Variables** | **Hazard Ratio** | **95% Confidence interval** | **Standard Error** |
| --- | --- | --- | --- |
| **Risk group^#^** |  | | |
| Vaccinated with no prior infection | 0.68 | 0.61 – 0.77 | 0.04 |
| Unvaccinated with prior infection | 0.14 | 0.09 – 0.23 | 0.04 |
| Vaccinated with prior infection | 0.09 | 0.05 – 0.16 | 0.03 |
| **Age** | 1.00 | 0.99 – 1.00 | 0.003 |
| **Gender** |  | | |
| Male | 0.95 | 0.83 – 1.09 | 0.06 |
| **Professional category*** |  | | |
| Attendant | 0.82 | 0.63-1.06 | 0.11 |
| Clerical staff | 1.03 | 0.70-1.53 | 0.21 |
| Nursing | 1.16 | 0.91-1.47 | 0.14 |
| Support staff | 1.03 | 0.80-1.31 | 0.13 |
| Pharmacist | 1.16 | 0.80-1.67 | 0.21 |
| Technicians | 0.93 | 0.70-1.22 | 0.13 |
| Trainee doctors | 1.00 | 0.76-1.32 | 0.14 |

^#^Unvaccinated with no prior infection is the reference group

*Consultant doctors are the reference group
