# Supplementary material for: Protective effect conferred by prior infection and vaccination on COVID-19 in a healthcare worker cohort in South India
Source: PLoS One. 2022 May 20;17(5):e0268797. doi: 10.1371/journal.pone.0268797 (PMC9122209; doi:10.1371/journal.pone.0268797)
Supplement: Attachment — Submitted filename: Response to the Reviewers R2.docx [file pone.0268797.s005.docx]

**Response to the Reviewers` Comments**

**Manuscript ID: PONE-D-21-35055**

**Reviewer #1:**

1. The data was not made available, NOT EVEN TO THE REVIEWERS because the link the authors provide is not working: when trying to access it I get "DOI Not Found" the error; let me be very clear about this: if the next time the link is still not working I will be rejecting the paper for good. Here is the link provided by authors : Dryad - <https://doi.org/10.5061/dryad.n8pk0p2x8>

**Response:**

The authors sincerely apologize for the trouble in the link. We have shared the private review link in the cover letter dated 18.02.2022. (<https://datadryad.org/stash/share/fNoszj0lxymYnzlkSzW004Gu16B8UYYReQn8yqM6GSo>) This dataset has been submitted to Dryad on 18.02.2022 and it was under the curation process. The DOI mentioned would have been activated only after acceptance of article, curation of the dataset and payment to the data repository. We have requested for an expedited data curation and we are happy to share that the data is now published on 17.04.2022. The variables which are considered as direct and indirect identifiers were removed as mandated by the data repository requirements. We are happy to share the entire dataset (excel) to the reviewer by email, if needed.

Link to access the dataset: <https://doi.org/10.5061/dryad.n8pk0p2x8>

2. There are severe limitations of the study because of the age homogeneity (everything concerns people with similar ages); these limitations should be VERY CLEARLY STATED : a) in the abstract, b) in the discussion section, c) when discussing the Cox regression, d) when discussing any generalization of the findings to another population.

**Response:**

The age distribution in our cohort varies mainly from 18-85 years of age which we feel is a fairly good distribution for HCW [Ref supplementary figure 1 which was provided on suggestion from the reviewer] and therefore we do not consider it as a severe limitation. The retirement age is 60 in our hospital and few employees contribute voluntary work after the retirement age (even upto 85 years). The age range is in keeping with other HCW studies in our country as we do have a younger work force when compared to western population (Eg SIREN study [1] which report a median age 45·7 years [IQR 35·4–53·5]).We do agree that median of 33.9 years of age does indicate a younger population but this is in keeping with the trend observed with COVID incidence in the general population in India (please note below)and hence we cannot categorise this as a severe limitation. Similar trends have been shown in studies published from India.

A study by Kushwaha et al., 2021 [2] showed that the mean age of all COVID-19 patients was 39.47 ± 17.59 years and the difference between the mean age of males (39.98 ± 17.19 years) and females (38.50 ± 18.29 years) in a cohort of 112,860 covid positive patients in India. Another study published by Chatterjee et al., 2020 [3] reported that the mean age of the cases was 34.73 yr [±standard deviation (SD): 9.64; median: 33.0; interquartile range (IQR): 27-40] in a health care worker cohort. Another study by Reddy et al., 2021 aimed at looking the age difference between the first and second wave of pandemic in India by analysing a total of 2,19,832 and 2,34,815 samples respectively [4]. The mean age during the first and the second wave were 35.1 ± 15.9 years and 46.1 ± 16.8 years respectively.

As suggested by the reviewer, we have clearly mentioned this as a possible limitation for generalization to the community at large in the “Limitations” section (Ref: page no 11; line number 287-289) and compared it with other studies in the discussion section (Ref: page no 10; line number 261-264). We do not think it merits mention in the abstract.

**References:**

1.	Hall VJ, Foulkes S, Charlett A, Atti A, Monk EJM, Simmons R, et al. SARS-CoV-2 infection rates of antibody-positive compared with antibody-negative health-care workers in England: a large, multicentre, prospective cohort study (SIREN). The Lancet. 2021;397: 1459–1469. doi:10.1016/S0140-6736(21)00675-9

2.	Kushwaha S, Khanna P, Rajagopal V, Kiran T. Biological attributes of age and gender variations in Indian COVID-19 cases: A retrospective data analysis. Clin Epidemiol Glob Health. 2021;11: 100788. doi:10.1016/j.cegh.2021.100788

3.	Chatterjee P, Anand T, Singh KhJ, Rasaily R, Singh R, Das S, et al. Healthcare workers & SARS-CoV-2 infection in India: A case-control investigation in the time of COVID-19. Indian J Med Res. 2020;151: 459–467. doi:10.4103/ijmr.IJMR_2234_20

4.	Reddy MM, Zaman K, Mishra SK, Yadav P, Kant R. Differences in age distribution in first and second waves of COVID-19 in eastern Uttar Pradesh, India. Diabetes Metab Syndr. 2021;15: 102327. doi:10.1016/j.dsx.2021.102327
